# Unravelling the Impact of the Genetic Variant rs1042058 within the TPL2 Risk Gene Locus on Molecular and Clinical Disease Course Patients with Inflammatory Bowel Disease

**DOI:** 10.3390/cells10123589

**Published:** 2021-12-20

**Authors:** Yasser Morsy, Nathalie Brillant, Yannick Franc, Michael Scharl, Marcin Wawrzyniak, on behalf of the Swiss IBD Cohort Study Group

**Affiliations:** 1 Department of Gastroenterology and Hepatology, University Hospital Zurich, University of Zurich, 8091 Zurich, Switzerland; yasser.morsy@usz.ch (Y.M.); Nathalie.DeBois-BrillantAndreu@usz.ch (N.B.); marcin.wawrzyniak@uzh.ch (M.W.); 2 Center for Primary Care and Public Health (Unisanté), University of Lausanne, 1101 Lausanne, Switzerland; yannick.franc@unisante.ch

**Keywords:** TPL2, IBD, ulcerative colitis, Crohn’s disease

## Abstract

**Background:** The single nucleotide polymorphism (SNP) rs1042058 within the gene locus encoding tumor progression locus 2 (TPL2) has been recently identified as a risk gene for inflammatory bowel disease (IBD). TPL2 has been shown to regulate pro-inflammatory signaling and cytokine secretion, while inhibition of TPL2 decreases intestinal inflammation in vivo. However, the clinical and molecular implications of this disease-associated TPL2 variation in IBD patients have not yet been studied. **Methods:** We analyzed the impact of the IBD-associated TPL2 variation using clinical data of 2145 genotyped patients from the Swiss IBD Cohort Study (SIBDCS). Furthermore, we assessed the molecular consequences of the TPL2 variation in ulcerative colitis (UC) and Crohn’s disease (CD) patients by real-time PCR and multiplex ELISA of colon biopsies or serum, respectively. **Results:** We found that presence of the SNP rs1042058 within the TPL2 gene locus results in significantly higher numbers of CD patients suffering from peripheral arthritis. In contrast, UC patients carrying this variant feature a lower risk for intestinal surgery. On a molecular level, the presence of the rs1042058 (GG) IBD-risk polymorphism in TPL2 was associated with decreased mRNA levels of IL-10 in CD patients and decreased levels of IL-18 in the intestine of UC patients. **Conclusions:** Our data suggest that the presence of the IBD-associated TPL2 variation might indicate a more severe disease course in CD patients. These results reveal a potential therapeutic target and demonstrate the relevance of the IBD-associated TPL2 SNP as a predictive biomarker in IBD.

## 1. Introduction

The single nucleotide polymorphism (SNP) rs1042058 within the gene encoding tumor progression locus 2 (TPL2) has recently been identified as an IBD risk gene [1]. On a functional level, TPL2 is involved in the regulation of innate immune cell and T-cell responses. In addition, it plays a critical role in controlling pathogen recognition receptor (PRR)-mediated cytokine secretion, such as TNF and IFNγ [2,3,4,5].

In specific, TPL2 has been shown to regulate pro-inflammatory signaling and cytokine secretion and has been demonstrated to play an essential role in mouse models of intestinal inflammation [6,7]. It actively controls the MAPK and NFκB pathway by phosphorylating MEK1, MEK2, MEK3, MEK6, JNK, ERK1/2, p38, and NFκB in response to PRR ligands, such as lipopolysaccharide (LPS) or muramyl dipeptide (MDP) [6,8].

Though mice lacking TPL2 exhibited normal NFκB signaling, they revealed attenuated colitis severity accompanied by reduced levels of pro-inflammatory cytokines, suggesting that the observed anti-inflammatory effects are likely to be mediated via MAPK-signaling rather than NFκB signaling [9]. In humans, Hedl et al. previously demonstrated that the homozygous (GG) IBD-associated variant within the TPL2 gene locus rs1042058 enhanced TPL2 mRNA and protein expression in human monocyte-derived macrophages derived from healthy individuals and Crohn’s disease (CD) patients. Presence of this genetic variant further resulted in enhanced NOD2-initiated phosphorylation of TPL2, ERK, JNK, and NFκβ as well as secretion of IL-1β and IL-18. These data suggested that the presence of the IBD-associated rs1042058 GG polymorphism can be considered a gain-of-function variant, as it enhances TPL2 expression and fosters TPL2-mediated signaling, finally promoting PRR-initiated events [8].

Those previous findings render TPL2 an exciting molecule for further investigation in IBD patients. However, to date, there are no data about the functional consequences of the presence of the IBD-associated TPL2 SNP in IBD patients in terms of intestinal and systemic cytokine levels. Similarly, the clinical relevance of this TPL2 SNP for the IBD disease course is yet unknown. Thus, we studied the impact of the IBD-associated TPL2 gene variation using patient samples and data from the Swiss IBD Cohort Study (SIBDCS) to address this knowledge gap. Using the clinical data of this large patient collective and a broad number of intestinal tissue and serum samples, we identified the relevance of the IBD-associated TPL2 variation for the disease course of IBD patients on a clinical and molecular level.

## 2. Materials and Methods

### 2.1. Patient Data and Cohort Design

Demographic and clinical patient data were obtained from the Swiss Inflammatory Bowel Disease Cohort Study (SIBDCS) database. The cohort includes patients with inflammatory bowel disease (IBD) throughout Switzerland as reported by their treating gastroenterologist. The medical data were collected annually. Additionally, blood samples and biopsies (in cases of colonoscopy), collected at baseline and during follow-up, were stored in a biobank [10,11]. The SIBDCS has been approved by the respective ethical committees in Switzerland (Ethics Committee of the Canton Zürich: EK-1316). All patients signed informed consent for data collection and analysis for research purposes before inclusion. The presented study has been evaluated and approved by the scientific board of the SIBDCS. Inflamed and uninflamed intestinal biopsies (n = 99) and serum samples (n = 157) were utilized from patients with the corresponding clinical data (Supplementary Table S1).

### 2.2. Genetic Analyses and Clinical Annotations

Genotyping for the presence of the IBD-associated SNP rs1042058 within the TPL2 gene locus was performed in 2145 SIBDCS patients using MALDI-TOFF mass spectrometry as described previously [12]. A total of 1197 patients had Crohn’s disease (CD), and 948 suffered from ulcerative colitis (UC) or indeterminate colitis (IC). Overall, 383 (17.9%) patients exhibited the TPL2 AA wild-type alleles, 1011 (47.1%) were heterozygous (AG), and 751 (35.0%) revealed the homozygous TPL2 variant alleles (GG). For statistical analyses, the AG and GG groups were merged to compare the “presence of the G-Allele” to its absence.

### 2.3. Real-Time RT-PCR and Western Blot

To avoid possible batch effect, analysis was performed at the same time, when samples from all patients were collected. For RNA isolation, biopsies were mechanically disrupted in 150 μL RLT buffer and dithiothreitol (Qiagen, Düsseldorf, Germany) using the gentleMACS tissue disrupter according to the manufacturer’s instructions. Total RNA was isolated using an RNeasy Mini Kit (Qiagen, Düsseldorf, Germany) and a QIA-Cube automated sample preparer (Qiagen). RNA concentration was measured by UV260-280 nm using a NanoDrop spectrophotometer (ND-1000; Witec AG, Sursee, Switzerland). A total of 1 µg RNA was transcribed to complementary DNA (cDNA) using the High-Capacity cDNA Reverse Transcription Kit (Applied Biosystems, Thermo Fisher Massachusetts, United states). Amplification steps were as follows: initial enzyme activation for 5 min at 95 °C, followed by 45 cycles of denaturing for 15 s at 95 °C and annealing/extending for 1 min at 60 °C each. Measurements were performed in triplicates using human GAPDH as endogenous control (VIC, MGB probe). The following TaqMan genotyping assays were obtained from Thermo Fisher (Waltham, MA, USA): Human TPL2 (Hs01566396_m1), IL-1 beta (Hs01555410_m1), IL-18 (Hs01038788_m1), TNIP2 (Hs01557654_mH), TNF alpha (Hs00174128_m1), IFN gamma (Hs00989291_m1), TGF beta (Hs00998133_m1), FOXP3 (Hs01085834_m1), IL-6 (Hs00174131_m1), IL-10 (Hs00961622_m1), IL-17 (Hs00174383_m1), DEFB4A (Hs00823638_m1), and GAPDH (4326317E) for mRNA quantification using TaqMan Fast Universal PCR Master Mix No AmpErase UNG (Applied Biosystems, Thermo Fisher, Waltham, MA, USA) on QuantStudio 6 Flex system.

Proteins were isolated from colon biopsies using mammalian protein extraction reagent (M-Per; Fisher Scientific, Waltham, MA, USA) according to the manufacturer’s instructions. After 30 min, lysates were centrifuged for 10 min at 17,000 *g* and protein-containing supernatants collected into fresh tubes. Each lysate was mixed with NuPAGE^®^ 4× LDS Sample Buffer (Life Technologies Ltd., Waltham, MA, USA) loading buffer, 500 mM dithiothreitol (DTT) and boiled for 5 min at 95 °C. Equal amounts of proteins from each lysate were loaded and separated on a 12% SDS-polyacrylamide gel electrophoresis (SDS-PAGE) and transferred onto nitrocellulose membranes for 1 h (Life Technologies Ltd. Massachusetts, United states). Membranes were blocked for 1 h at room temperature (RT) with 5% bovine serum albumin (BSA) in Tris-buffered saline containing 1% Tween (TBST) prior to overnight incubation at 4 °C with the following antibodies: anti-MAP3K8/COT (1:500; 52, 53 kDa) (ab137589), anti-Phospho-TPL2 (1:500; 60, 62 kDa) (PA5-36635), anti-phospho-SAPK/JNK (1:1000; 54 kDa) (9251S), anti-SAPK/JNK (1:1000, 54 kDa) (9252S), anti-phospho-p44/42 MARK (1:1000; 42, 44 kDa) (9101S) and anti-p44/42 MAPK (1:1000; 42, 44 kDa) (9102S), anti-phospho-NF-kappa B p65 (1:1000; 65 kDa) (3033S), and anti-NF-κB p65 (1:1000; 65 kDa) (8242S), anti-IL1-b (1:1000; 17, 30 kDa) (12703), anti-phospho-p38 MAPK(1:1000; 38 kDa) (9216S), anti-p38 MAPK (1:1000; 38 kDa) (9212S) anti-b-actin (1:20000; 43 kDa)( MABT523). After washing 30 min (3 times for 10 min) in TBST, membranes were incubated with Horseradish peroxidase (HRP)-labelled secondary anti-mouse- or anti-rabbit-IgG-antibody (1:2000; Santa Cruz Biotechnologies, Dallas, TX, United states) for 1 h. Immunoreactive proteins were detected with a Fusion Solo S imager (Vilber Lourmat; Witec AG Lucerne, Switzerland) using a WesternBright ECL-HRP Substrate (#K-12045-D50; Witec AG Lucerne, Switzerland). Densitometric analysis of Western blots was performed using the image J software.

### 2.4. Multiplex Assays of Cytokines in Human Serum

The quantification of 27 cytokines, chemokines and growth factors in human serum samples from CD and UC patients were analyzed by Bio-Plex Pro™ Human Cytokine 27-plex Assay (#M500KCAF0Y, Bio-Rad Laboratory, Hercules, CA, USA) according to the pre-optimized protocol based on the methodology provided by the manufacturer. The complete list of cytokines, chemokines and growth factors detected by the Bio-Rad multiplex microbead assays and their assay characteristics (https://www.bio-rad.com/en-ch/sku/m500kcaf0y-bio-plex-pro-human-cytokine-27-plex-assay?ID=m500kcaf0y accessed on 20 October 2021). Data were collected and analyzed using a Bio-Rad BioPlex 200 instrument equipped with Bio-Plex Manager software version 6.0 (Bio-Rad Laboratory, Hercules, CA, USA). The precision based on both intra- and inter-assays variations were <15% and <25%, respectively, within the detection limits provided by the manufacturer. The immunoassay data were expressed in terms of pg/mL.

### 2.5. Statistical Analysis

Statistical analyses were performed using the statistical program Stata (version 16.0, College Station, TX, USA). Categorical data are summarized as frequencies and percentages. For quantitative data, differences between the two groups were evaluated by using Mann–Whitney tests. For categorical data, differences in observed frequencies between groups were tested by using the chi-squared test or the exact Fisher test in case of small sample size (n ≤ 5). For the present study, a *p*-value < 0.05 is considered statistically significant. Hierarchical clustering (package pheatmap v1.0.12), correlation (package Hmisc v4.6-0) and multi-dimensional factorial (package FactoMineR v2.4) analysis were performed using R version 4.1.0.

## 3. Results

Among all tested patients, 1197 patients had Crohn’s disease (CD), and 948 suffered from ulcerative colitis (UC) or indeterminate colitis (IC). Overall, 383 (17.9%) patients exhibited the TPL2 AA wild-type alleles, 1011 (47.1%) were heterozygous (AG), and 751 (35.0%) revealed the homozygous TPL2 variant alleles (GG). For statistical analyses, the AG and GG groups were merged to compare the “presence of the G-Allele” to its absence.

### 3.1. The Presence of the TPL2 Risk Allele Is Associated with Peripheral Arthritis in CD Patients

We studied the clinical characteristics of 1197 CD patients obtained from the SIBDCS regarding the presence of the TPL2 risk allele. In univariate analysis, patients carrying the risk allele (AG or GG) had a significantly higher risk of developing peripheral arthritis than patients carrying wild-type alleles (*p* = 0.005, Table 1). However, according to the Montreal classification, neither the initial or current disease location nor the onset of fistulas, anal fissures, abscesses, or stenosis differed significantly between genotypes. In addition, the onset of complications and the use or response/non-response to medications did not differ between genotypes (Supplementary Table S2).

### 3.2. The Presence of the TPL2 Risk Allele Is Associated with Fewer Numbers of Intestinal Surgeries in UC Patients

Next, we studied the clinical characteristics of the 948 UC/IC patients obtained from the SIBDCS concerning the presence of the TPL2 risk allele. Interestingly, patients carrying the risk allele (AG or GG) required significantly fewer intestinal surgeries than patients carrying the wild-type alleles (*p* = 0.022), albeit the number of patients who underwent surgery overall was relatively small (Table 1). However, there were no significant differences between genotypes in initial or current disease location (pancolitis, left-sided colitis, proctitis) or the onset of complications (considering the very small n for deep venous thrombosis), extraintestinal manifestations, or use of or response/non-response to medications (Supplementary Table S3).

### 3.3. The Presence of TPL2 Genotypes Had a Low Impact on mRNA Levels of Some Cytokines in IBD Patients

**Figure 1:**
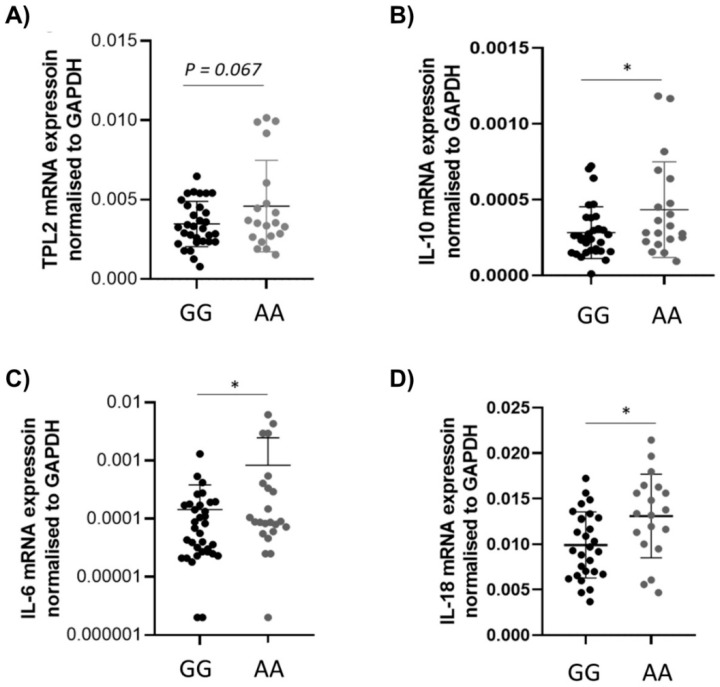
Cytokines IL-10 and IL-6, and IL-18 are decreased in patients with rs1042058 TPL2 variant in CD and UC, respectively. Intestinal biopsies from CD and UC patients with or without rs1042058 TPL2 variant were analyzed for mRNA expression of inflammatory cytokines. Graphs demonstrate mRNA levels of (**A**) TPL2 in CD patients stratified by the presence of the rs1042058 TPL2 variant, (**B**) IL-10 in DC patients, (**C**) IL-6 in CD patients, and (**D**) IL-18 mRNA expression in UC patients shows a significant decrease when carrying the rs1042058 TPL2 variant. Error bars represent means ± SD. * = *p* < 0.05.

To investigate the activation pathway of TPL2 in SNP carriers and non-carriers, assessment of mRNA expression of different cytokines was performed using qRT-PCR, according to standard protocols [13]. A total of 99 inflamed intestinal biopsies from CD (n = 52) and UC (n = 47) patients with or without the rs1042058 TPL2 variant were used. The levels of TPL2 mRNA were reduced in CD or UC patients, although this effect was not statistically significant (Figure 1A). On the other hand, the mRNA expression of the anti-inflammatory cytokine IL-10 as well as of the pro-inflammatory IL-6 were significantly decreased in CD patients carrying the TPL2 variant (Figure 1B,C). Additionally, the mRNA level of the cytokine IL-18 was significantly decreased in UC patients carrying the TPL2 variant (Figure 1D). All other studied cytokines (TNF-α, IFN-γ, TGF-β, IL-1β, IL-17) and genes (TNIP, Foxp3, DEF4B) did not show significant differences (data not shown). Additionally, the correlative analysis of Tpl2 expression vs. cytokines in the wild type and the variant did not show significant differences in either UC or CD (Supplementary Figure S1).

**Figure 2:**
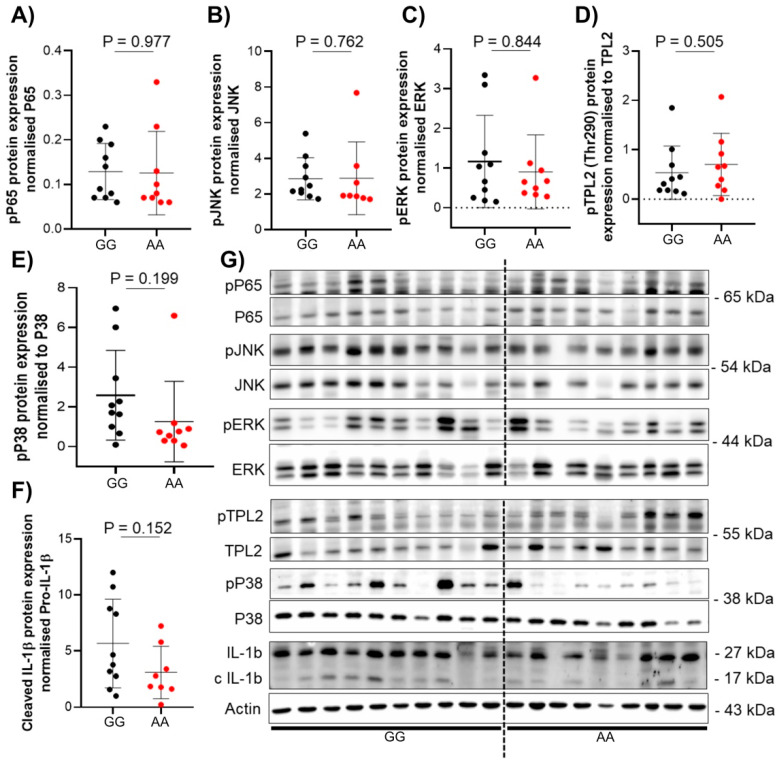
Protein expression of TPL2 downstream pathway in CD patient with or without rs1042058 TPL2 variant. Analysis of protein expression in human colon biopsies was performed using western blot from CD patients with or without rs1042058 TPL2 variant. (**A**–**F**) Dots in the graph represent individual patients. Red circles represent patients with the rs1042058 TPL2 variant, and black circles represent patients without the rs1042058 TPL2 variant. (**G**) Each lane in the Western blot image represents individual patients. Error bars represent means ± SD.

**Figure 3:**
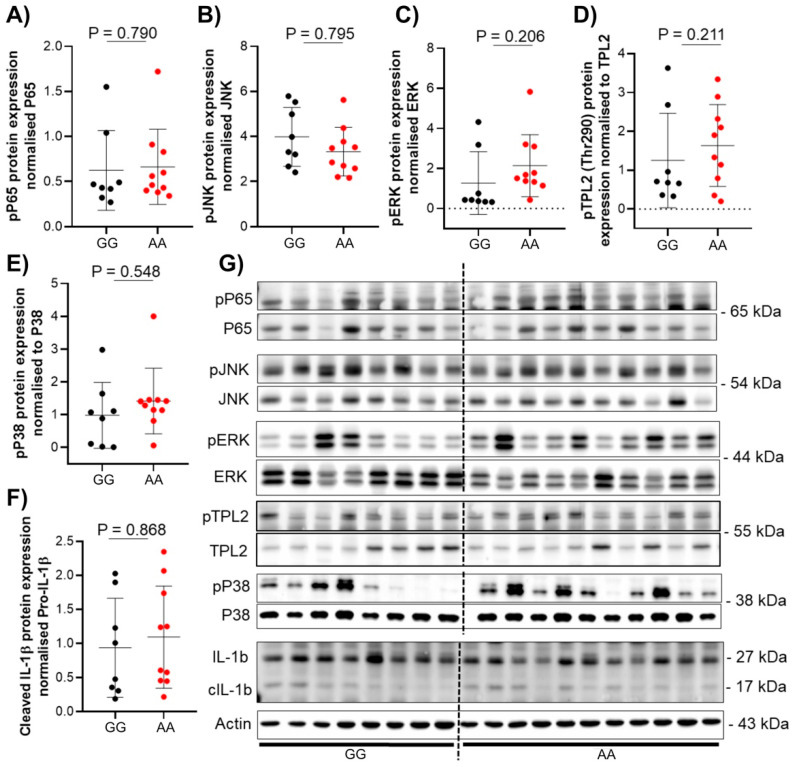
Protein expression of TPL2 downstream pathway in UC patients with or without rs1042058 TPL2 variant. Analysis of protein expression in human colon biopsies was performed using western blot from UC patients with or without rs1042058 TPL2 variant. (**A**–**F**) Dots in the graph represent individual patients. Red circles represent patients with the rs1042058 TPL2 variant, and black circles represent patients without the rs1042058 TPL2 variant. (**G**) Each lane in the Western blot image represents an individual patient. Error bars represent means ± SD.

To investigate the phenotypical differences in patients carrying the TPL2 variant compared to non-carriers, protein analysis of TPL2 downstream pathway activation was performed from colon biopsies (n = 46) of either CD (n = 20) or UC (n = 26) patients. Protein expression was assessed using Western blot according to standard protocols [13]. No statistical differences in protein expression related to genotype or disease state were seen in either CD or UC patient samples (Figure 2 and Figure 3).

### 3.4. The Presence of TPL2 Risk Alleles Has No Impact on Serum Cytokine Levels in IBD Patients

**Figure 4:**
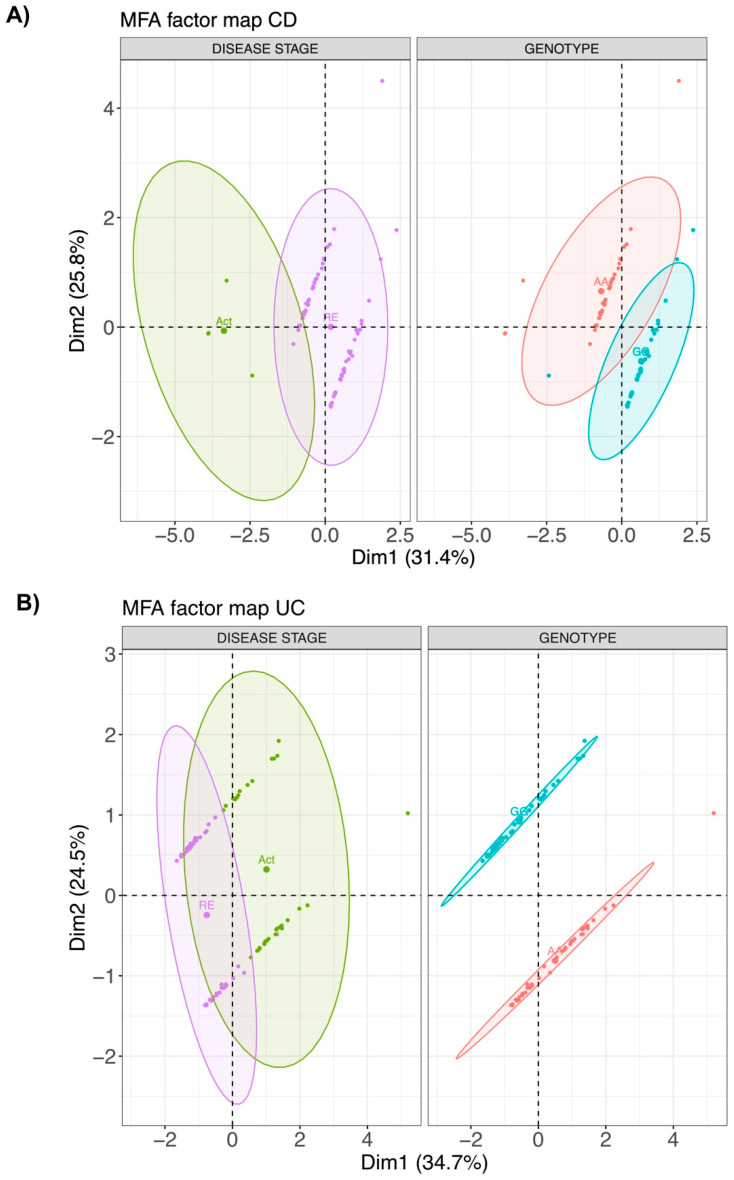
Multi-factorial analysis (MFA) of 27 cytokines measured in serum from both CD (**A**) and UC (**B**) patients. Levels of various cytokines were determined in blood samples from CD and UC patients in active (Act) or remission (RE) state in both wild-type and variant using ELISA.

**Figure 5:**
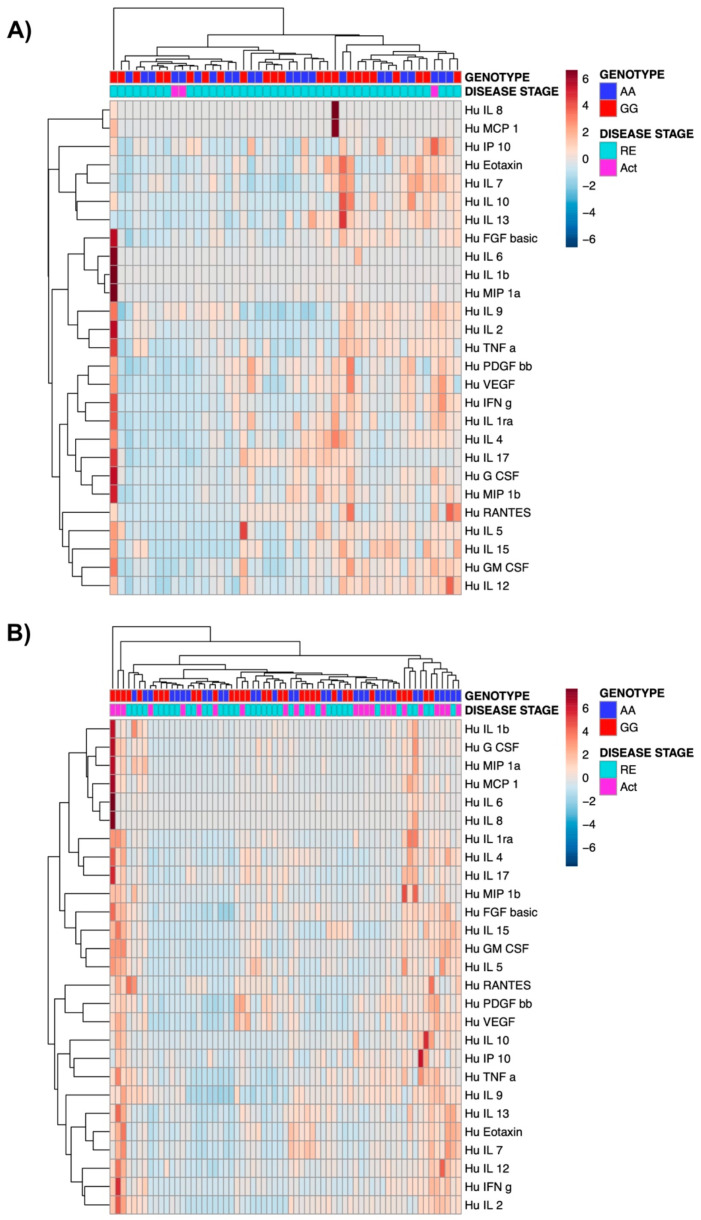
Heatmaps of 27 cytokines measured in serum from both CD (**A**) and UC (**B**) patients. Levels of various cytokines were determined in blood samples from CD and UC patients in active (Act) or remission (RE) state in both wild type and variant using ELISA.

We next studied whether the presence of the TPL2 risk alleles might affect the composition of serum cytokine levels in IBD patients. For serum cytokine analysis, serum samples (n = 155) from patients with CD (n = 76) and UC (n = 79) were utilized. We detected a clustering based on PCA analyses based on genotype and on disease activity in both UC and CD patients (Figure 4). However, none of the comparisons between detected cytokines were statistically significant between patients carrying the TPL2 risk allele vs. wild-type patients. Further, the hierarchical clustering did not show a clear cluster as shown in the respective heatmaps (Figure 5).

## 4. Discussion

IBD is an immune-mediated disease characterized by dysregulated cytokine secretion. CD and UC are distinct chronic relapsing and remitting inflammatory disorders of the bowel. They share key features in their pathogenesis, such as excessive innate and adaptive immune responses, a dysfunctional intestinal epithelial barrier, and alterations in the microbiota [14,15,16,17,18,19]. Interestingly, different mRNA cytokine levels were observed in biopsies from patients carrying the TPL2 variant. Decreased IL-18 mRNA levels in biopsies from UC patients carrying the TPL2 variant and decreased IL-10 and IL-6 mRNA levels in biopsies from CD patients carrying the TPL2 variant were observed compared to wild-type patients. In addition to immunological and environmental factors, genetic predisposition is a significant risk factor for the development of IBD [20]

Here, we show that the presence of the rs1042058 GG IBD-risk polymorphism within the TPL2 gene locus results in decreased IL-10, IL-6, and IL-18 mRNA levels in the intestine of CD and UC patients, respectively. However, although our data demonstrated the association between the TPL2 variant with reduced mRNA levels of the anti-inflammatory IL-10 and pro-inflammatory IL-6 cytokines in the intestine of CD patients, we did not find an impact on the protein level of cytokines or signaling proteins. Previously published data suggested that the presence of the IBD-associated rs1042058 GG polymorphism can be considered a gain-of-function variant, as it enhanced TPL2 mRNA and protein expression in human monocyte-derived macrophages derived from healthy individuals and Crohn’s disease (CD) patients. Presence of this genetic variant further resulted in enhanced NOD2-initiated phosphorylation of TPL2, ERK, JNK and NFκβ as well as secretion of IL-1β and IL-18 [8]. However, reported results were obtained from experiments performed in vitro using differentiated cells, and therefore lack the comprehensiveness of multi-layer analysis applicable to more complex multicellular tissue. Additionally, TPL2 might be differently regulated in mononuclear cells in blood compared to tissue cells.

Our data suggest that the presence of the respective SNP is associated with a more severe disease course in CD patients as indicated by more severe extraintestinal manifestations (EIM); however, the data also suggest less risk for colectomy in UC patients. It is generally accepted that CD and UC have different pathogenetic mechanisms, and thus a functionally relevant SNP in an IBD risk gene, namely in the TPL2 gene, might have different implications on the disease course. Since the pathogenesis of EIM in IBD, particularly CD patients, is also still only poorly understood, aberrant expression and/or function of TPL2 in CD patients might, in the end, have a protective function, while in UC patients, it might contribute to a more severe disease course. Our data suggest that the TPL2 function is not specific for CD or UC. Since TPL2 is a general regulator of inflammation, it may possibly be involved in both IBD as well as *Clostridium difficile*-induced intestinal inflammation. Overall, this is the first study reporting the rs1042058 polymorphism of TPL2 in IBD on both the clinical and molecular levels.

In our study, multi-dimensional factorial analysis of cytokines in serum showed that patients carrying the TPL2 variant were separated from wild-type patients based on total cytokine composition. In addition, the analysis could also separate patients based on the disease stage, showing a clear separation between patients with active disease vs. patients in remission. However, on a single cytokine level, we did not observe statistically significant differences between CD or UC patients carrying TPL2 risk allele as compared to wild-type patients.

In summary, we found that the presence of the SNP rs1042058 within the TPL2 gene locus results in a significantly higher number of CD patients suffering from peripheral arthritis accompanied by lower levels of the anti-inflammatory cytokine IL-10. Thus, our findings suggest that presence of the rs1042058 SNP within the TPL2 gene could serve as a potential biomarker in CD patients. However, its possible use as a biomarker in clinical practice would need further prospective validation.

## Figures and Tables

**Table 1:** Clinical characteristics of CD and UC patients according to presence of TPL2 risk alleles.

Genotype (CD)	AA	AG	GG
Number (%)	210 (17.5)	562 (47)	425 (35.5)
CD patients	AA	AG/GG	*p*-value (Wilcoxon)
Age at Diagnosis			
Median, q25–q75,	23, 17–31,	23.5, 17–34,	0.657
min–max	5–73	1–81	
Genotype:	AA	AG/GG	*p*-value (chi^2^)
Gender			
Men	110 (52.4)	491 (49.7)	0.488
Women	100 (47.6)	496 (50.3)	
Peripheral arthritis			
Yes	21 (14.8)	232 (23.5)	0.005
No	179 (85.2)	755 (76.5)	
Uveitis/Iritis			
Yes	1 (0.5)	10 (1)	0.459
No	209 (99.5)	977 (99)	
Pyoderma gangrenosum			
Yes	1 (0.5)	5 (0.5)	0.955
No	209 (99.5)	982 (99.5)	
Erythema nodosum			
Yes	0	8 (0.8)	0.191
No	210 (100)	979 (99.2)	
Aphtous oral ulcers			
Yes	6 (2.9)	27 (2.7)	0.922
No	204 (97.1)	960 (97.3)	
Ankylosing spondylitis			
Yes	6 (2.9)	14 (1.4)	0.140
No	204 (97.1)	973 (98.6)	
Prim. scler. cholangitis			
Yes	0	5 (0.5)	0.301
No	210 (100)	982 (99.5)	
Other			
Yes	2 (0.9)	10 (1)	0.936
No	208 (99.1)	977 (99)	
Summary of Extraintestinal manifestations			
Yes	40 (19)	258 (26.1)	0.031
No	170 (81)	729 (73.9)	
**Genotype (UC):**	**AA**	**AG**	**GG**
Number (%)	173 (18.3)	449 (47.4)	326 (34.3)
UC patients	AA	AG or GG	*p*-value (Wilcoxon)
Age at Diagnosis			
Median, q25–q75,	26, 19–37,	27, 17–36,	0.404
min–max	3–75	2–79	
Number (%)	AA	AG or GG	*p*-value (chi^2^)
Gender			
Men	81 (46.8)	426 (55)	0.052
Women	92 (53.2)	349 (45)	
Surgery			
Yes	7 (4)	11 (1.4)	0.022
No	166 (96)	764 (98.6)	

## Data Availability

The data underlying this article will be shared on reasonable request to the corresponding author.

## Supplementary Materials

The following are available online at https://www.mdpi.com/article/10.3390/cells10123589/s1. Figure S1. Correlation analysis of TPL2 variant expression with mRNA cytokines expression from both CD and UC patients. Table S1. Clinical and phenotypical characteristics of Swiss IBD cohort patients with CD and UC with (GG) or without (AA) rs1042058 TPL2 variant in numbers. Table S2. Clinical characteristics of CD patients according to presence of TPL2 risk alleles. Table S3. Clinical characteristics of UC patients according to presence of TPL2 risk alleles.
