# Supplementary material for: Unravelling the Impact of the Genetic Variant rs1042058 within the TPL2 Risk Gene Locus on Molecular and Clinical Disease Course Patients with Inflammatory Bowel Disease
Source: Cells. 2021 Dec 20;10(12):3589. doi: 10.3390/cells10123589 (PMC8700574; doi:10.3390/cells10123589)
Supplement: Supplementary file 1 [file cells-10-03589-s001.zip › cells-1489280-supplementary.pdf]

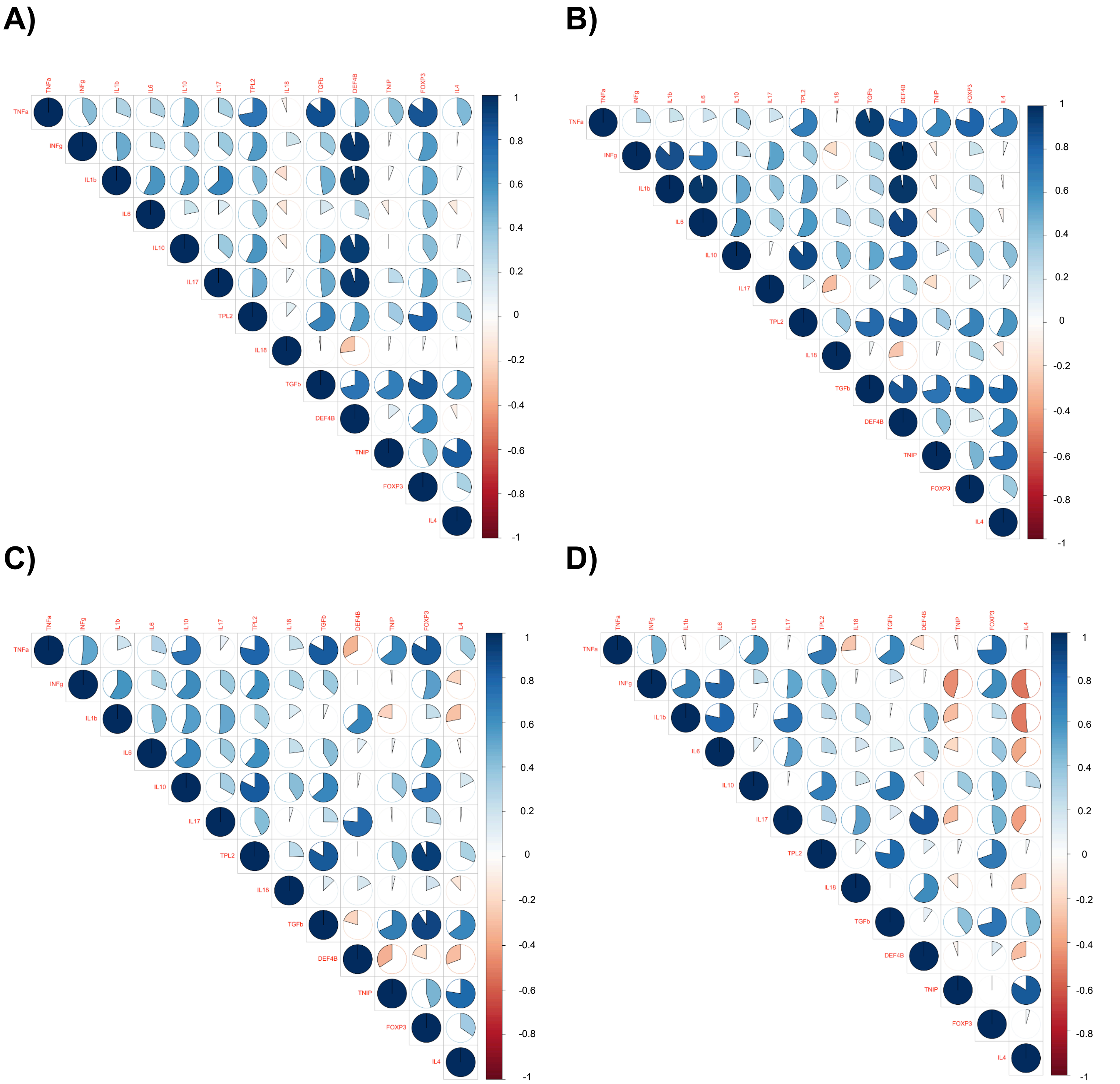

Supplementary Figure S1. Correlation analysis of TPL2 variant expression with mRNA cytokines expression from both CD and UC patients.

Correlation analysis between TPL2 and cytokines in both CD with variant rs1042058 (A) and CD wild type (B),in addition to UC with variant (C) and wild type (D), blue color indicate positive correlation and red color represents negative regulation.

Supplementary Table S1.

Clinical and phenotypical characteristics of Swiss IBD cohort patients with CD and UC with (GG) or without (AA) rs1042058 TPL2 variant in numbers

Clinical characteristics of patients		CD		UC	
		GG	AA	GG	AA
Number of patients for analysis	Number of biopsies for protein analysis	10	9	14	12
	Number of biopsies for mRNA analysis	32	20	28	19
	Number of blood samples cytokine analysis	39	37	37	42
Disease activity index	Patients in complete remission (CDAI < 150, MTWAI < 3)	50	48	31	30
	Patients with active disease (CDAI > 150, MTWAI > 3)	5	4	20	25
Genetic Background	Patients with or without rs1042058 TPL2 variant	55	52	51	55
Montreal classification	B1	31	33	50	53
	B2	15	8	0	1
	B3	9	10	1	1
	"p" perianal disease	12	10	2	0
	NA	0	1	0	0
Intestinal surgery	patients with intestinal surgery	22	20	10	6
Immunosupressive	Patients with immunosuppressive treatment successful	32	30	29	31
	Patients with immunosuppressive treatment failure	40	28	38	33
Steroids	Patients with steroids treatment successful	51	65	57	46
	Patients with steroids treatment failure	14	13	13	10
Stenosis		38	27	3	3
Fistula		58	54	6	8
Extraintestinal manifestations		60	52	41	39

Supplementary Table S2.

Clinical characteristics of CD patients according to presence of TPL2 risk alleles.

Number (%)	AA	AG or GG	p-value (chi2)
**Initial disease location**			
L1	44 (20.9)	206 (20.9)	0.517
L2	32 (15.2)	166 (16.8)	
L3	89 (42.4)	417 (42.3)	
L4	3 (1.4)	8 (0.8)	
L1 + L4	0	17 (1.7)	
L2 + L4	4 (1.9)	10 (1)	
L3 + L4	15 (7.1)	70 (7.1)	
Unknown	23 (10.9)	93 (9.4)	
**Current disease location**			
L1	54 (25.7)	*265 (26.8)*	0.499
L2	65 (30.9)	*301 (30.5)*	
L3	57 (27.1)	*286 (29)*	
L4	6 (2.8)	*29 (2.9)*	
L1 + L4	3 (1.4)	*21 (2.1)*	
L2 + L4	8 (3.8)	*14 (1.4)*	
L3 + L4	12 (5.7)	*52 (5.3)*	
Unknown	5 (2.4)	*19 (1.9)*	
**Perianal fistula**			
Yes	14 (6.7)	54 (5.5)	0.497
No	196 (93.3)	933 (94.5)	
**Other fistula**			
Yes	8 (3.8)	32 (3.2)	0.678
No	202 (96.2)	955 (96.8)	
**Anal fissure**			
Yes	6 (2.9)	24 (2.4)	0.720
No	204 (97.1)	963 (95.6)	
**Abscess**			
Yes	6 (2.9)	47 (4.8)	0.223
No	204 (97.1)	940 (95.2)	
**Summary of History of fistula**			
Yes	31 (14.8)	118 (12)	0.263
No	179 (85.2)	869 (88)	
**Stenosis**			
Yes	25 (11.9)	112 (11.4)	0.818
No	185 (88.1)	875 (88.6)	
**Intestinal Surgery**			
Yes	7 (3.3)	35 (3.5)	0.879
No	203 (96.7)	952 (96.5)	

Supplementary Table S2. continuation

Clinical characteristics of CD patients according to presence of TPL2 risk alleles.

Number (%)	AA	AG or GG	p-value (chi2)
**Osteopenia (–porosis)**			
Yes	9 (4.3)	63 (6.4)	0.246
No	201 (95.7)	924 (93.6)	
**Deep Venous Thrombosis**			
Yes	0	0	
No	210 (100)	987 (100)	
**Pulmonary Embolism**			0.514
Yes	0	2 (0.2)	
No	210 (100)	985 (99.8)	
**Gallstone**			0.695
Yes	1 (0.5)	3 (0.3)	
No	209 (99.5)	984 (99.7)	
**Nephrolithiasis**			0.955
Yes	1 (0.5)	5 (0.5)	
No	209 (99.5)	982 (99.5)	
**Malabsorption syndrome**			0.936
Yes	2 (0.9)	10 (1)	
No	208 (99.1)	977 (99)	
**Perforation / Peritonitis**			0.885
Yes	1 (0.5)	4 (0.4)	
No	209 (99.5)	983 (99.6)	
**Adv. Effect of Treatment**			0.257
Yes	0	6 (0.6)	
No	210 (100)	981 (99.4)	
**Summary of Complications**			0.118
Yes	12 (5.7)	89 (9)	
No	198 (94.3)	898 (91)	
**Use of Anti-TNF**			
Yes	107 (51)	474 (48)	0.441
No	103 (49)	513 (52)	
**Failure or non-response to Anti-TNF therapy**			
Yes	21 (55.3)	75 (48.7)	0.469
No	17 (44.7)	79 (51.3)	
**Use of Steroids**			
Yes	71 (33.8)	312 (31.6)	0.535
No	139 (66.2)	675 (68.4)	
**Non-response to steroids**			
Yes	17 (38.6)	60 (37)	0.846
No	27 (61.4)	102 (63)	
**Number of follow-ups with a therapy with steroids**			
0	26 (12.4)	131 (13.3)	0.679
1	67 (31.9)	302 (30.6)	
2	37 (17.6)	188 (19)	
3	30 (14.3)	118 (12)	
4	16 (7.6)	82 (8.3)	
5	9 (4.3)	49 (5)	
6	7 (3.3)	39 (4)	
7	3 (1.4)	21 (2.1)	
8	5 (2.4)	19 (1.9)	
9	4 (1.9)	17 (1.7)	
10	0	12 (1.2)	
11	3 (1.4)	5 (0.5)	
12	2 (0.9)	3 (0.3)	
13	1 (0.5)	1 (0.1)	

Supplementary Table S2. continuation

Clinical characteristics of CD patients according to presence of TPL2 risk alleles.

Number (%)	AA	AG or GG	p-value (chi2)
**Use of Azathioprine**			
Yes	48 (22.9)	238 (24.1)	0.981
No	162 (77.1)	749 (75.9)	
**Use of 6-Mercaptopurine**			
Yes	5 (2.4)	29 (2.9)	0.659
No	205 (97.6)	958 (97.1)	
**Use of Antibiotics**			
Yes	13 (6.2)	90 (9.1)	0.169
No	197 (93.8)	897 (90.9)	
**Use of Methotrexate**			
Yes	18 (8.6)	95 (9.6)	0.635
No	192 (91.4)	892 (90.4)	
**Use of Cyclosporine**			
Yes	0	1 (0.1)	0.644
No	210 (100)	986 (99.9)	
**Use of Tacrolimus**			
Yes	1 (0.5)	0	0.175
No	209 (99.5)	987 (100)	
**Summary of these therapies**			
Yes	19 (9)	96 (9.7)	
No	191 (91)	891 (90.3	0.762

Supplementary Table S3.

Clinical characteristics of UC patients according to presence of TPL2 risk alleles.

Number (%)	AA	AG or GG	p-value (chi^2)
**Initial disease location**			
Pancolitis	*78 (45)*	*335 (43.2)*	0.975
Left-sided Colitis	*50 (28.9)*	*233 (30.1)*	
Proctitis	*25 (14.4)*	*117 (15.1)*	
Unknown	*20 (11.6)*	*90 (11.6)*	
**Current disease location**			
Pancolitis	*80 (46.2)*	*354 (45.7)*	0.734
Left-sided Colitis	*55 (31.8)*	*270 (34.8)*	
Proctitis	*34 (19.6)*	*129 (16.6)*	
Unknown	*4 (2.3)*	*22 (2.8)*	
**Osteopenia (–porosis)**			
Yes	14 (8.1)	39 (5)	0.113
No	159 (91.9)	736 (95)	
**Deep Venous Thrombosis**			**0.033**
Yes	2 (1.2)	0	
No	171 (98.8)	775 (100)	
**Pulmonary Embolism**			0.245
Yes	1 (0.6)	1 (0.1)	
No	172 (99.4)	774 (99.9)	
**Gallstone**			0.344
Yes	0	4 (0.5)	
No	173 (100)	771 (99.5)	
**Nephrolithiasis**			0.726
Yes	1 (0.6)	3 (0.4)	
No	172 (99.4)	772 (99.6)	
**Malabsorption syndrome**			0.078
Yes	3 (1.7)	3 (0.4)	
No	170 (98.3)	772 (99.6)	
**Perforation / Peritonitis**			
Yes	0	0	
No	173 (100)	775 (100)	
**Adv. Effect of Treatment**			0.919
Yes	1 (0.6)	4 (0.5)	
No	172 (99.4)	771 (99.5)	
**Summary of Complications**			0.054
Yes	19 (11)	52 (6.7)	
No	154 (89)	723 (93.3)	
**Peripheral arthritis**			
Yes	19 (11)	86 (11.1)	0.966
No	154 (89)	689 (88.9)	
**Uveitis / Iritis**			
Yes	1 (0.6)	3 (0.4)	0.726
No	172 (99.4)	772 (99.6)	
**Pyoderma gangrenosum**			
Yes	1 (0.6)	0	0.182
No	172 (99.4)	775 (100)	
**Erythema nodosum**			
Yes	0	4 (0.5)	0.446
No	173 (100)	771 (99.5)	

Supplementary Table S3. continuation

Clinical characteristics of UC patients according to presence of TPL2 risk alleles.

Number (%)	AA	AG or GG	p-value (chi^2)
**Aphtous oral ulcers**			
Yes	1 (0.6)	17 (2.2)	0.223
No	172 (99.4)	758 (97.8)	
**Ankylosing spondylitis**			
Yes	3 (1.7)	7 (0.9)	0.333
No	170 (98.3)	768 (99.1)	
**Prim. scler. cholangitis**			
Yes	1 (0.6)	17 (2.2)	0.159
No	172 (99.4)	758 (97.8)	
**Other**			
Yes	2 (1.2)	8 (1)	0.885
No	171 (98.8)	767 (99)	
**Summary of Extraintestinal manifestations**			
Yes	24 (13.9)	127 (16.4)	0.414
No	149 (86.1)	648 (83.6)	
**Use of Anti-TNF**			
Yes	46 (26.6)	190 (24.5)	0.568
No	127 (73.4)	585 (75.5)	
**Failure or non-response to Anti-TNF therapy**			
Yes	11 (45.8)	44 (55)	0.430
No	13 (54.2)	36 (45)	
**Use of Steroids**			
Yes	62 (35.8)	248 (32)	0.331
No	111 (64.2)	527 (68)	
**Non-response to steroids**			
Yes	13 (35.1)	44 (34.1)	0.908
No	24 (64.9)	85 (65.9)	
**Use of Azathioprine**			
Yes	41 (23.7)	181 (23.4)	0.923
No	132 (76.3)	594 (76.6)	
**Use of 6-Mercaptopurine**			
Yes	7 (4)	25 (3.2)	0.589
No	166 (96)	750 (96.8)	
**Use of Antibiotics**			
Yes	10 (5.8)	54 (7)	0.574
No	163 (94.2)	721 (93)	
**Use of Methotrexate**			
Yes	8 (4.6)	40 (5.2)	0.771
No	165 (95.4)	735 (94.8)	
**Use of Cyclosporine**			
Yes	1 (0.6)	5 (0.7)	0.920
No	172 (99.4)	770 (99.3)	
**Use of Tacrolimus**			
Yes	3 (1.7)	16 (2.1)	0.779
No	170 (98.3)	759 (97.9)	
**Summary of these therapies**			
Yes	12 (7)	60 (7.7)	0.718
No	161 (93)	715 (92.3)	
